# The extravasation of contrast as a predictor of cerebral hemorrhagic contusion expansion, poor neurological outcome and mortality after traumatic brain injury: A systematic review and meta-analysis

**DOI:** 10.1371/journal.pone.0235561

**Published:** 2020-07-07

**Authors:** Isabella Vargas Baldon, Andre Candeas Amorim, Larissa Marques Santana, Davi J. Solla, Angelos Kolias, Peter Hutchinson, Wellingson S. Paiva, Marcos Rosa-Júnior

**Affiliations:** 1 Department of Radiology, Hospital Universitário Cassiano Antônio de Moraes da Universidade Federal do Espírito Santo – HUCAM/UFES/EBSERH, Vitória, State of Espírito Santo, Brazil; 2 Department of Neurology, Division of Neurosurgery, Hospital das Clínicas of the University of São Paulo, São Paulo, Brazil; 3 NIHR Global Health Research Group on Neurotrauma, University of Cambridge, Cambridge, United Kingdom; 4 Division of Neurosurgery, Department of Clinical Neurosciences, Addenbrooke’s, Hospital and University of Cambridge, Cambridge, United Kingdom; 5 Department of Neuroradiology, Hospital Universitário Cassiano Antônio de Moraes da Universidade Federal do Espírito Santo – HUCAM/UFES/EBSERH, Vitória, State of Espírito Santo, Brazil; George Washington University, UNITED STATES

## Abstract

**Background:**

The active extravasation of contrast on CT angiography (CTA) in primary intracerebral hemorrhages (ICH) is recognized as a predictive factor for ICH expansion, unfavorable outcomes and mortality. However, few studies have been conducted on the setting of traumatic brain injury (TBI).

**Purpose:**

To perform a literature systematic review and meta-analysis of the association of contrast extravasation on cerebral hemorrhagic contusion expansion, neurological outcomes and mortality.

**Data sources:**

The PubMed, Cochrane Library, Medline, Scielo, VHL and IBECS databases up to September 21, 2019, were searched for eligible studies.

**Study selection:**

A total of 505 individual titles and abstracts were identified and screened. A total of 36 were selected for full text analysis, out of which 4 fulfilled all inclusion and exclusion criteria.

**Data analysis:**

All 4 studies yielded point estimates suggestive of higher risk for hematoma expansion with contrast extravasation and the summary RR was 5.75 (95%CI 2.74–10.47, p<0.001). Contrast extravasation was also associated with worse neurological outcomes (RR 3.25, 95%CI 2.24–4.73, p<0.001) and higher mortality (RR 2.77, 95%CI 1.03–7.47, p = 0.04).

**Data synthesis:**

This study is a Systematic Review and Meta-Analysis revealed the extravasation of contrast is a useful imaging sign to predict hematoma expansion, worse neurological outcomes and higher mortality.

**Limitations:**

Only four articles were selected.

**Conclusions:**

The extravasation of contrast in the setting of TBI is a useful imaging sign to predict hematoma expansion, worse neurological outcomes and higher mortality.

## Introduction

Traumatic brain injury (TBI) is the leading cause of death in victims of trauma and may results in the development of intra-axial contusions, of which hematoma is a key component. Long-term neurological deficits are reported in approximately half of the TBI survivors and this type of injury is often also responsible for cognitive and behavioral deficits [1–5].

It should be noted that up to 65% of the patients with cerebral contusion have hemorrhagic expansion when evaluated with serial computed tomography (CT) [6]. During the acute phase, significant hematoma expansion can be associated with neurological decline, need for surgery and higher mortality [7–14]. The survivors are predisposed to less successful rehabilitation and unfavorable long-term outcomes [14–17].

Some variables have been studied and are potential predictors of cerebral hemorrhagic contusion hematoma: patient age, initial hematoma volume, acute subdural hematoma, traumatic subarachnoid hemorrhage, midline shift, decompressive craniectomy, time delay from TBI to admission head CT, need for cardiopulmonary resuscitation, hyperglycemia and coagulopathy (either by previous antiplatelet / anticoagulant use or trauma induced coagulopathy) [6–8, 10, 13–15, 18–23].

The active extravasation of contrast on CT angiography (CTA) has been extensively studied in spontaneous intracerebral hemorrhage (ICH) and is recognized as a predictive factor for ICH expansion, unfavorable outcomes and mortality [19, 24–32]. However, few studies have been conducted on the impact of this variable in the setting of TBI. In this study, we performed a systematic review and meta-analysis of the association of contrast extravasation on cerebral hemorrhagic contusion expansion, neurological outcomes and mortality.

## Materials and methods

### Protocol and registration

This systematic review was conducted following the criteria of the Preferred Reporting Items for Systematic Review and Meta-Analyzes (PRISMA) [33]. The protocol was registered in Prospero’s database (International Prospective Register of Systematic Reviews) by the number CRD42020102723. The study was approved by the University Hospital Cassiano Antônio Moraes ethics committee. The consent term was not obtained because the data was analyzed anonymously.

### Eligibility criteria

We included prospective or retrospective studies, in English, Spanish or Portuguese language that evaluated the effect of extravasation of contrast on hematoma expansion, neurological outcome and/or mortality. We excluded articles with animal experiments, non-contrast CT, non-traumatic intracerebral hematoma, iatrogenic brain hemorrhages, extra-axial (subdural or extradural) hematomas, subarachnoid hemorrhage, intraventricular hemorrhage and case reports. Studies where the only imaging modality was Magnetic Resonance Imaging (MRI) were also excluded.

### Database searched

The following databases were searched on September 21th, 2019: Pubmed, Scielo, Cochrane Library, Medline, Virtual Health Library and IBECS.

### Search strategy

This meta-analysis was conducted following the PRISMA statement [33]. The following search strategy was employed in English: (spot sign OR blush sign OR contrast extravasation OR leakage sign OR postcontrast leakage) AND (traumatic brain contusion OR traumatic brain injury OR traumatic brain hematoma OR cerebral hematoma OR intracranial hematoma OR brain hematoma OR traumatic intracranial hematoma OR traumatic cerebral hematoma OR traumatic cerebral contusion OR traumatic intracranial contusion OR traumatic cerebral injury OR traumatic intracranial injury OR brain contusion hematoma OR intracranial contusion hematoma OR cerebral contusion hematoma).

### Study selection

Two reviewers (AFCA and IVB) screened the titles and abstracts to identify eligible articles. Duplicate articles were removed with the support of EndNote software and abstracts that did not provide enough data were retained for full-text analysis. Independent reviewers performed the full-text analysis to judged which articles were to be included. Disagreements were resolved by consensus and, when necessary, the opinion of a third reviewer (MRJ) was considered. Furthermore, we manually searched the reference list of the selected articles for additional relevant studies and performed citation analysis through Google Scholar.

### Risk of bias and quality assessment

The selected papers were critically assessed by the QUADAS-2 tool to evaluate the risk of bias [34].

### Data extraction

Two reviewers (AFCA and IVB) reviewed the full text of the selected papers and extracted data in standardized and independent forms. The following items were extracted: author, year, study design, location, sample size, gender, imaging modality, CT type, definition of hemorrhage expansion and assessment blinding. Since there is a variation in the definition of hematoma expansion in the literature (range 5–12 milliliters and >30–33%), we accepted all definitions cited in the articles. Raw data were extracted into 2x2 contingency tables of positive and negative contrast extravasation against hematoma expansion, neurological outcome and mortality.

### Statistical analysis

Risk ratios (RR) and 95% confidence intervals (CI) were used as summary statistics for the binary outcome hematoma expansion, neurological outcome and mortality. The studies were integrated with the Mantel-Haenszel method for random-effects, based on the inverse-variance approach for calculating the weights. Statistical heterogeneity was quantified with the I2 statistics for descriptive purposes. We decided to use the more conservative random-effects approach even though statistical heterogeneity was not significant due to qualitative heterogeneity between the studies. The presence of publication bias was planned to be assessed by visual inspection of funnel plots and by the Egger regression asymmetry test. A two-tailed p value < 0.05 was considered significant. Data analysis was performed using the software Review Manager (RevMan, version 5.3).

## Results

**Fig 1:**
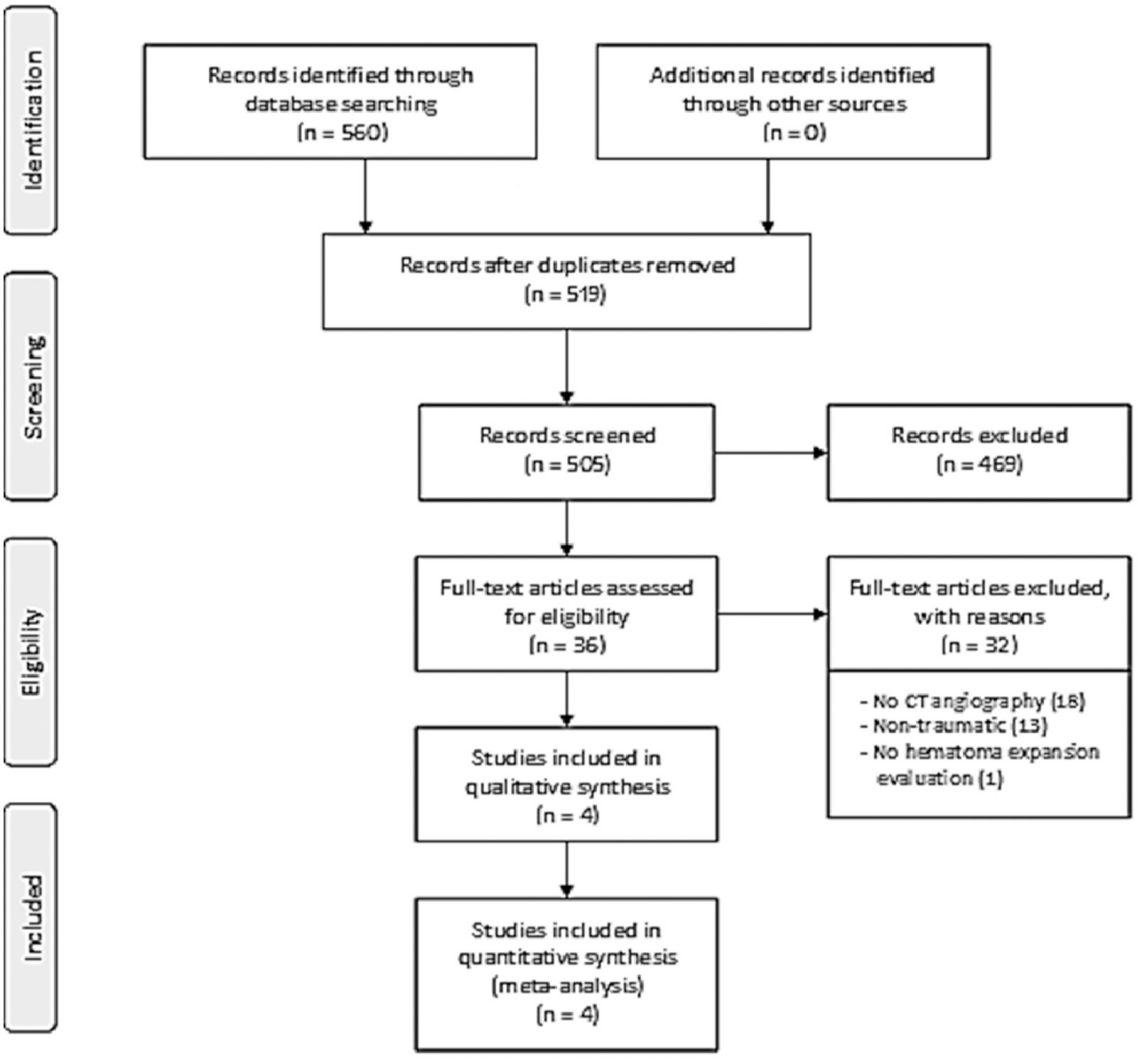
Systematic review flowchart.

We identified 505 individual titles and abstracts for screening. A total 36 were selected for full text review, of which 4 satisfied all inclusion and exclusion criteria. The reasons for exclusion were: non-CTA (18), non-traumatic (13) and absence of hematoma expansion evaluation (1). Fig 1 outlines the flowchart of the systematic review [33].

**Fig 2:**
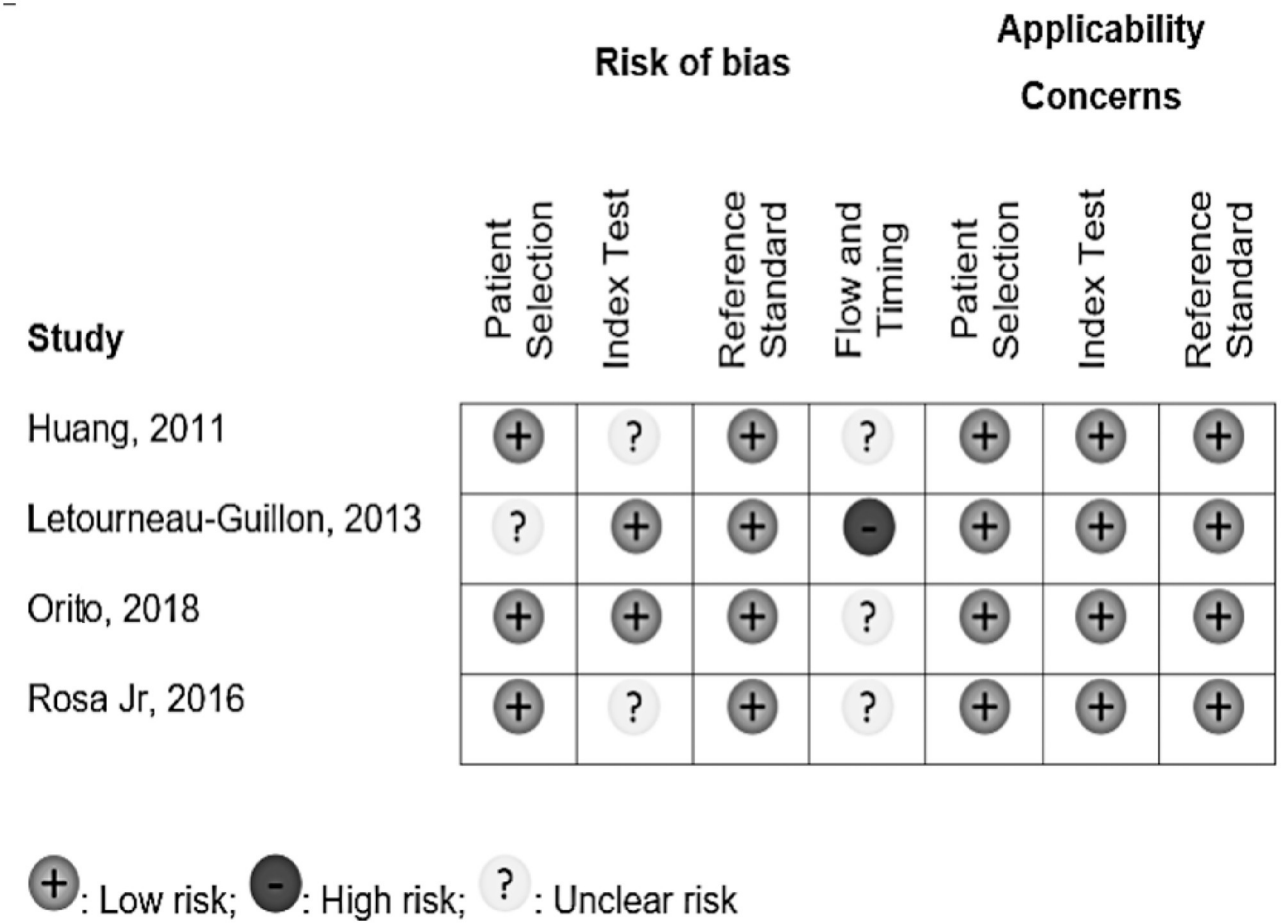
Risk of bias summary for the included studies.

The quality assessment of the included studies is presented on Fig 2.

The study by Huang et al. [35] in 2011 was the first approaching this thematic in the trauma scenario. They assessed 22 patients with traumatic brain contusion diagnosed by CT scan. Contrasted-enhanced CT was performed 6 hours after the trauma. Hemorrhage progression was defined as a greater than 30% or more than 5 mL increase in the contusion hematoma volume on CT performed at the time of neurologic deterioration or on follow-up CT at 24 hours or 72 hours. The contrast extravasation was identified in 41% of the patients (9/22) and was significantly associated with hemorrhage expansion, worse neurological outcomes and the need for surgical intervention.

The second study was published in 2013 by Letourneau-Guillon et al. [36] They aimed to assess the prognostic value of contrast extravasation in traumatic intracranial hematomas. Hemorrhage progression was defined as an increase greater than 12 mL and 33%. They included 60 patients, of which 50% (30/60) presented contrast extravasation. Contrast extravasation was associated with greater initial total hematoma volume, midline shift larger than 5mm, hematoma expansion, worse neurological outcomes and higher risk of death.

The next study was developed by Rosa Jr et al. in 2016 [37]. On a cohort of 121 patients, they investigated whether the contrast extravasation was a predictor of hematoma expansion (available on 96 patients), poor outcomes and mortality in traumatic brain hemorrhagic lesions. Expansion of the hemorrhagic component of the contusion was defined at the follow-up CT by an absolute growth greater than 6mL or a relative growth of more than 33% from the initial CT. Expansion of the hematoma was observed in 61.1% (22/36) of the patients with contrast extravasation, while only 10% (6/60) of those without contrast extravasation presented hematoma expansion. Contrast extravasation was also significantly associated with worse neurological outcomes and higher risk of death.

The last study included in this systematic review was published in 2018 by Orito et al. [38]. They included a total of 33 patients, but emergency hematoma evacuation at admission was performed in 11 of these, nine of whom had extravasation of contrast. Delayed hematoma evacuation was performed later in four of the remaining 22 patients, all of whom had extravasation of contrast. Any hematoma expansion was considered. Analyzing these 22 patients not submitted to surgery on admission, they concluded that contrast extravasation could predict traumatic hematoma expansion and the necessity of surgical intervention.

**Fig 3:**
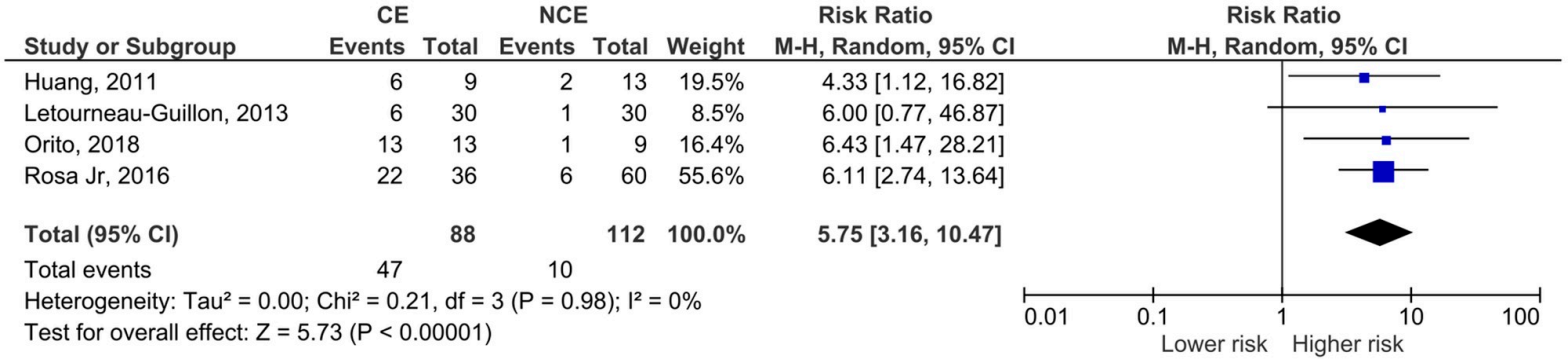
Meta-analysis for the effect of computed tomography angiography contrast extravasation on traumatic contusion expansion.

In summary, for the outcome of hematoma expansion, all four studies were included. All of them had yielded point estimates suggestive of higher risk for hematoma expansion with contrast extravasation (Table 1). The summary RR was 5.75 (95% CI 2.74–10.47), p<0.001 (Fig 3).

**Table 1:** Summarizes the characteristics of the included studies.

Author, year	Study design	Location	Sample size	Male	Imaging modality	CT type	Definition of HE	Blinded assessment
Orito (2018) [38]	Prospective	Japan	22	72.7%	First-pass CTA; second pass PCCT	8-section	Any HE	Yes
Rosa Jr (2016) [37]	Prospective	Brazil	121	87.6%	First-pass CTA; second pass PCCT	64-section	>6ml or >33%	NA
Letourneau-Guillon (2013) [36]	Retrospective	Canada	60	77.0%	First-pass CTA; second pass PCCT	64-section	>12ml and >33%	Yes
Huang (2011) [35]	Prospective	Taiwan	22	63.6%	First-pass CTA; second pass PCCT	NA	>5ml or >30%	NA

CT: computed tomography; HE: Hematoma expansion; CTA: CT angiography; PCCT: postcontrast CT; NA: not available.

**Fig 4:**
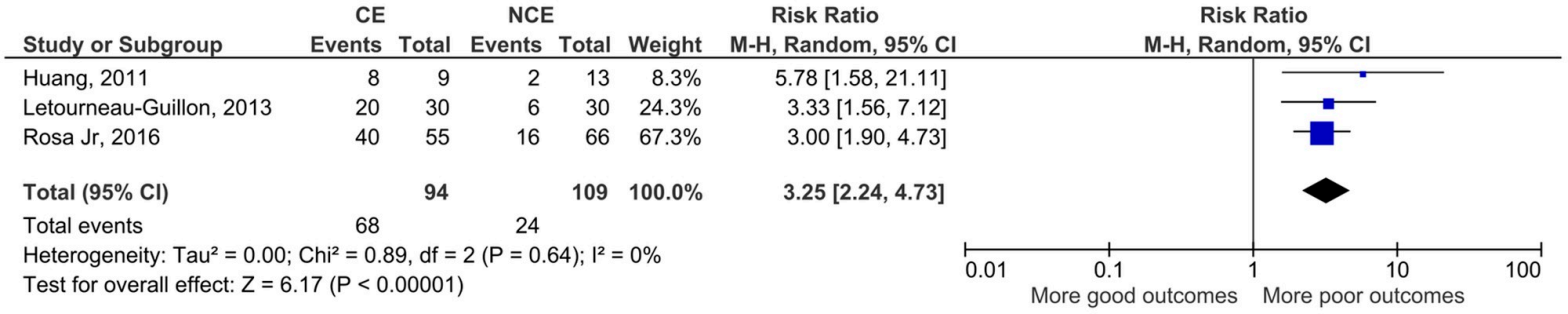
Meta-analysis for the association between computed tomography angiography contrast extravasation on traumatic contusion and neurological outcomes.

**Fig 5:**
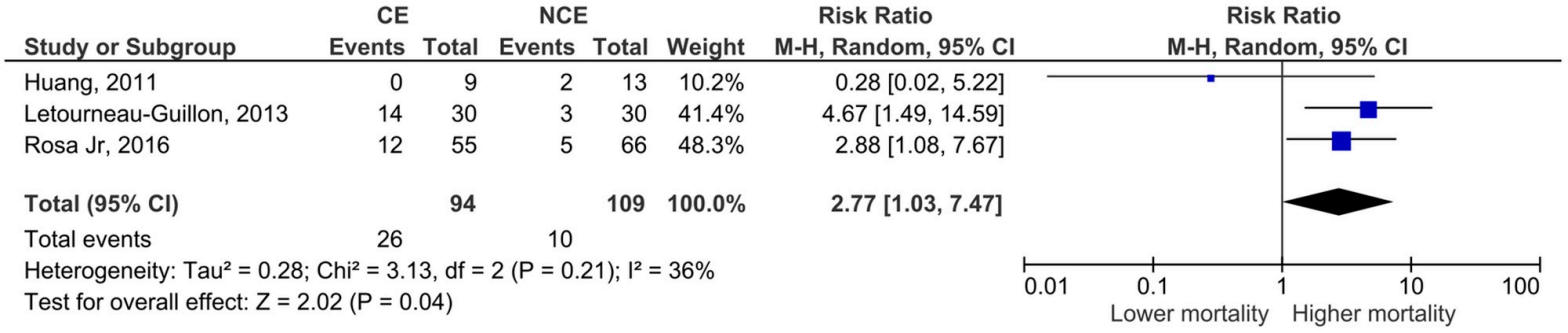
Meta-analysis for the association between computed tomography angiography contrast extravasation on traumatic contusion and mortality.

Regarding neurological outcomes (Fig 4) and mortality (Fig 5), the study by Orito et al. could not be included (only descriptive data was presented for these outcomes, without stratification according to contrast extravasation).

Contrast extravasation was associated with worse neurological outcomes (RR 3.25, 95% CI 2.24–4.73, p<0.001) and higher mortality (RR 2.77, 95% CI 1.03–7.47, p = 0.04).

The presence of publication bias was planned to be assessed by visual inspection of funnel plots and by the Egger regression asymmetry test, but due to the low number of eligible studies (less than 10) this method was not suitable. In this situation, the power of the tests is too low to distinguish chance from real asymmetry [39].

## Discussion

TBI is the leading cause of death following trauma. Furthermore, survivors frequently have neurological disabilities, some of them for their whole life. Approximately 38–65% of the patients with traumatic brain injuries develop hematoma expansion [7, 38, 40], which worsens the patients´ prognosis since it might lead to increased intracranial pressure (ICP) [6].

The biological processes observed on the acute stage of traumatic brain hemorrhages are somewhat like those observed in primary hemorrhagic stroke. The progression of hemorrhage causes secondary injury in the surrounding brain parenchyma, can increase mass effect, elevate intracranial pressure, and initiate pathologic cascades which ultimately result in further injury. Studies using Xenon-enhanced CT revealed that cerebral contusions present pericontusional zones of low regional cerebral blood flow and edema, resembling the penumbral zone in the acute ischemic stroke. Thus, the perihematomal and pericontusional areas present considerable risk of secondary ischemic events [35, 41, 42].

Although some variables have been assessed as possible predictors of traumatic cerebral hemorrhagic contusion expansion, there are important limitations on their predictive performance [8, 10, 11]. Therefore, there exists a significant knowledge gap in this area of interest. A small number of studies have assessed the role of contrast extravasation in TBI, which is in contrast with a relatively large literature focused on spontaneous ICH. One possible explanation for this is the fact that many trauma centers do not routinely use contrast enhanced imaging for patients with TBI. This might explain why we only found four eligible articles for inclusion on our meta-analysis.

In the study of Orito, the angiography was realized during the initial CT scan, while 5 min after the initial scan a delayed-phase CT scan was performed. After this, a plain CT image was obtained 24 hours after the initial CT scan to asses the size of the hematoma. In the study of Rosa Jr, the imaging assessment in all patients using a CT scan adding a CTA to the initial requested NCCT. The control CT was realized by the clinical judgment, using just a NCCT in the first 3 days of admission in the Hospital. In the study of Huang the patients underwent first the NCCT and after that they realized CTA scan within 6 hours of the trauma as part of their admission CT survey. In the study of Letourneau-Guillon, the consecutive patients with head trauma with cerebral lesion of ≥10mm in the largest transverse diameter or acute extraaxial hematoma of ≥2 mm in thickness and with CTA performed within 24 hours of admission and with follow-up head CT in 72 hours of admission were included.

All the studies showed RR bigger than 1 in the hematoma expansion, neurological outcomes and mortality for exception of Huang that revealed a RR of 0.28 and 95% IC [0.02–5.22] in the mortality analysis. However, this data of the Huang study is not statistically significant in base of the 95% IC. Furthermore, the Huang study doesn’t focus on the mortality analysis.

Although the limited number and heterogeneity of the studies preclude conclusive statements, we believe that our study adds to the current available literature. The included articles reported much larger confidence intervals or even non-significant results. Thus, this meta-analysis contributes to reduce uncertainty and increase the likelihood of the hypothesis that contrast extravasation on CTA is associated with hematoma expansion, neurological outcomes and mortality in TBI patients. This hypothesis was based on spontaneous ICH studies. A recent meta-analysis by Xu et al in spontaneous ICH have shown that the spot-sign in first-pass CTA is associated with hematoma expansion (OR 8.5), poor neurological outcome (OR 6.4) and higher risk of 3-month mortality (OR 3.8) [43]. Another meta-analysis by Du et al also demonstrated the association of the spot-sign in different CT modalities with hematoma expansion (OR 8.7 to 52.6) in spontaneous ICH [44]. These effect sizes are not so different from the ones we show in Figs 3 to 5—the risk ratios presented convert to the following OR: hematoma expansion, OR 14,3 (4,8–41,5); neurological outcome, OR 9,2 (4,8–17,5); mortality, OR 3,2 (0,9–12,4).

This is the first meta-analysis in the medical literature addressing this question in the trauma scenario and provides support for further research in this area. Future studies could aim to define the indications for CTA following TBI and examine additional endpoints, such as need for neurosurgical intervention or ICP-lowering treatments. Robust prediction of hematoma expansion could also pave the way for studies of pharmacological agents that could counteract mechanisms underpinning hematoma expansion.

The main limitation of this study is the small number of eligible studies. This reflects the lack of scientific literature on the specific field (rather than a methodological issue), but it weakens the statistical robustness of the findings. Moreover, all four studies were single-center, with heterogenous definitions of hematoma expansion and neurological outcomes, which is restricting the generalizability of the results. Even so, the total number of patients (around two-hundred, depending on the outcome), is not negligible and the existing evidence in spontaneous ICH supports the biological plausibility of our findings. As new studies approaching this theme become available, further meta-analyses of aggregate data may advance our knowledge. Another limitation is that CTA is not universally available, so our findings are more relevant for well-developed heath care systems.

## Conclusion

The extravasation of contrast in the setting of traumatic brain contusion is a useful imaging sign to predict hematoma expansion, worse neurological outcomes and higher mortality. Considering the low number of studies and limited sample sizes, additional multi-center, adequately powered studies with more standardized methods are needed to the validation of this hypothesis and wider use, if appropriate.

## Supporting information

S1 Check list (DOC)
