# Correction: The extravasation of contrast as a predictor of cerebral hemorrhagic contusion expansion, poor neurological outcome and mortality after traumatic brain injury: A systematic review and meta-analysis

**DOI:** 10.1371/journal.pone.0238968

**Published:** 2020-09-03

**Authors:** Isabella Vargas Baldon, Andre Candeas Amorim, Larissa Marques Santana, Davi J. Solla, Angelos Kolias, Peter Hutchinson, Wellingson S. Paiva, Marcos Rosa-Júnior

There are errors in the Funding statement. The correct Funding statement is as follows: This research was partially funded by the National Council for Scientific and Technological Development (CNPq), Brazil. Dr Amorim was supported by CNPq, Brazil. Drs Solla, Kolias, Hutchinson and Paiva are supported by the NIHR Global Health Research Group on Neurotrauma, which was commissioned by the National Institute for Health Research (NIHR) using UK aid from the UK Government (project 16/137/105). The views expressed in this publication are those of the author(s) and not necessarily those of the NIHR or the Department of Health and Social Care. Angelos Kolias is supported by a Clinical Lectureship, School of Clinical Medicine, University of Cambridge and the Royal College of Surgeons of England. Peter Hutchinson is supported by a Research Professorship from the NIHR, the NIHR Cambridge Biomedical Research Centre, a European Union Seventh Framework Program grant (CENTER-TBI; grant no. 602150), and the Royal College of Surgeons of England.

## References

[1] Baldon IV, Amorim AC, Santana LM, Solla DJ, Kolias A, Hutchinson P, et al (2020) The extravasation of contrast as a predictor of cerebral hemorrhagic contusion expansion, poor neurological outcome and mortality after traumatic brain injury: A systematic review and meta-analysis. PLoS ONE 15(7): e0235561 10.1371/journal.pone.0235561 32634141 PMC7340282

